# Dynamics of Persistent and Acute Deformed Wing Virus Infections in Honey Bees, *Apis mellifera*

**DOI:** 10.3390/v3122425

**Published:** 2011-12-14

**Authors:** Gennaro Di Prisco, Xuan Zhang, Francesco Pennacchio, Emilio Caprio, Jilian Li, Jay D. Evans, Gloria DeGrandi-Hoffman, Michele Hamilton, Yan Ping Chen

**Affiliations:** 1 Dipartimento di Entomologia e Zoologia Agraria “Filippo Silvestri”, Universita’ degli Studi di Napoli “Federico II”, Via Universita’ n.100, 80055 Portici, Napoli, Italy; Email: gennaro.diprisco@unina.it (G.D.P.); f.pennacchio@unina.it (F.P.); emcaprio@unina.it (E.C.); 2 College of Plant Protection, Yunnan Agricultural University, Yunnan 650201, China; Email: zhang.xuan.yn@gmail.com; 3 Institute of Apicultural Research, Chinese Academy of Agricultural Science, Xiangshan, Beijing 100093, China; Email: bumblebeeljl@hotmail.com; 4 USDA-ARS Bee Research Laboratory, Beltsville, MD 20705, USA; Email: jay.evans@ars.usda.gov (J.D.E.); michele.hamilton@ars.usda.gov (M.H.); 5 USDA-ARS Carl Hayden Bee Research Center, Tucson, AZ 85719, USA; Email: Gloria.Hoffman@ars.usda.gov

**Keywords:** bee, viruses, *Varroa*, vitellogenin, temperature

## Abstract

The dynamics of viruses are critical to our understanding of disease pathogenesis. Using honey bee *Deformed wing virus* (DWV) as a model, we conducted field and laboratory studies to investigate the roles of abiotic and biotic stress factors as well as host health conditions in dynamics of virus replication in honey bees. The results showed that temperature decline could lead to not only significant decrease in the rate for pupae to emerge as adult bees, but also an increased severity of the virus infection in emerged bees, partly explaining the high levels of winter losses of managed honey bees, *Apis mellifera*, around the world. By experimentally exposing adult bees with variable levels of parasitic mite *Varroa destructor*, we showed that the severity of DWV infection was positively correlated with the density and time period of *Varroa* mite infestation, confirming the role of *Varroa* mites in virus transmission and activation in honey bees. Further, we showed that host conditions have a significant impact on the outcome of DWV infection as bees that originate from strong colonies resist DWV infection and replication significantly better than bee originating from weak colonies. The information obtained from this study has important implications for enhancing our understanding of host‑pathogen interactions and can be used to develop effective disease control strategies for honey bees.

## 1. Introduction

The European honey bee, *Apis mellifera L.* (*Hymenoptera: Apidae*), plays a vital role in agro‑ecosystems by assisting in the pollination of one-third of the world’s food crops. While there is a continuously increasing need for bees for crop pollination, the existing populations of honey bees in most parts of the world have undergone marked declines since the 1940s [1], threatening global agricultural production. An especially alarming phenomenon is the honey bee Colony Collapse Disorder (CCD), an event that wiped out large numbers of honey bees across the United States and around the world in 2006–2007 [2,3]. A survey of microbes in CCD-affected honey bee colonies showed a higher incidence of viruses [4,5,6], raising serious concerns about risks of virus infections and resulting in an urgent need for effective control strategies against viral diseases to safeguard the health of honey bees.

So far honey bees have been reported to be attacked by at least 19 viruses [7,8]. Except for the filamentous bee virus [9], iridescent virus [10], and Chronic bee paralysis virus [11], all honey bee viruses reported so far contain a single stranded RNA genome encapsulated in a non-enveloped icosahedral capsid with a diameter of 20–30 nm. Among honey bee viruses, *Deformed wing virus* (DWV) is one of the most common and prevalent viruses infecting honey bees [3,12]. This virus, originally isolated from diseased adult bees in Japan [13], has a worldwide distribution and has been reported in Europe, North America, South America, Africa, Asia, and the Middle East [7,8,14]. DWV contains a positive-sense, polyadenylated, and monopartite monocistronic RNA genome encoding for a large polyprotein with structural capsid proteins residing at the N-terminal section of the polyprotein and nonstructural proteins arranged in the order of a RNA helicase, a chymotrypsin-like 3C protease and an RNA-dependent RNA polymerase (RdRp) residing at the C-terminal section of the polyprotein. Based mainly on its virion structure and genome organization, DWV has been assigned to the genus, *Iflavirus*, family *Iflaviridae* [15,16]. 

DWV infection is among the most common viral infections in honey bees and the virus has established a persistent infection in most apiaries in the world. Infection by DWV was found in different bee castes including queen, drones, and workers as well as in different bee developmental stages including eggs, larvae, pupae and adults [17]. While DWV usually persists as an asymptomatic infection transmitted orally between adults through trophallaxis and vertically from mother queens to offspring in bee colonies [18,19,20,21], the virus can be re-activated at any time as a result of various host stress triggers, causing symptoms of illness to emerge in infected bees. The manifestations of the disease caused by DWV infection include shrunken, crumpled wings, decreased body size, discoloration in adult bees, and reduction in life span [13,22,23]. The severe symptoms of DWV infections are associated with infestations of the parasitic mite, *Varroa destructor*. Both laboratory and field studies showed that the *Varroa* mite is not only an effective vector of DWV by acquiring the virus from infected bees and transmitting it horizontally to uninfected bees during feeding [24,25,26,27,28,29,30,31], but also an activator of the virus by causing host immunosuppression and thereby fostering viral replication in bees [32]. Observation of strikingly high rates of DWV infection in association with *Varroa* mites suggests that DWV is a significant factor for bee mortality and colony losses. Therefore, there is a critical need to understand more fully the mechanisms responsible for inducing overt disease in inapparently or latently DWV-infected bees.

Viruses have evolved persistent and acute life strategies in hosts that can change populations through disease-induced mortality. The dynamics of viral replication, in turn, are dependent on the conditions of the hosts that are regulated by biotic and abiotic factors singly or in combination. The levels of viral disease expression in honey bees likely result from complex molecular and cellular interactions between the host and the pathogen. In order to gain more insight into the incidence of persistent DWV infection in honey bees, we conducted a field screening to investigate the abundance and seasonal changes of DWV in bees under natural conditions. Using DWV as a model pathogen, we also conducted laboratory experiments to investigate the dynamics of the virus replication in honey bees following stressful manipulations. The effects of abiotic and biotic factors on the DWV replication were assessed by (1) experimentally incubating sealed brood at different temperatures and comparing the DWV levels in newly emerged bees incubated either at optimal growth temperature or temperature that was cooler than normal condition, and (2) experimentally challenging newly emerged adult bees originating from strong and weak bee colonies with different levels of *Varroa* mites and comparing the DWV titer in different treatment groups. In addition, gene expression levels of vitellogenin (Vg), a precursor of egg yolk protein that is a plausible marker of general robustness and nutritional status of honey bees, were compared in bees from both strong and weak colonies to examine the correlation between the host health status and the virus titer in honey bees. 

## 2. Results and Discussion

### 2.1. Persistent Infection of DWV in Honey Bees

**Figure 1:**
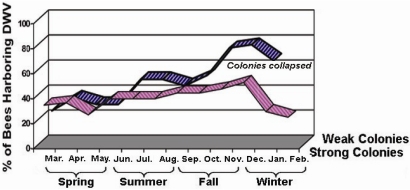
Seasonal activities of Deformed Wing Virus (DWV) infection in strong and weak honey bee colonies. DWV infection was detected in every month of the year but the infection rates varied from month to monthduring the study. All weak colonies died in January before the completion of the survey.

DWV has established a persistent infection within honeybee hosts in nature. While the vast majority of the DWV infected bees appeared to be asymptomatic, DWV infection was found in every apiary investigated and in every month of the year during the study. The percentage of pooled DWV infection rate was 25% and 35% for strong colonies and weak colonies, respectively, in the first month of the study (Figure 1). The infection rate began to rise after the spring and reached a peak of 53% and 77.5% for strong colonies and weak colonies, respectively, in the early winter month of December before declining by the end of winter. Although there was no statistically significant difference in DWV infection rates between the strong and weak colonies during the spring, the infection of DWV was detected more frequently in weak colonies than in strong colonies after spring. The infection rate of DWV in bees originating from weak colonies was significantly higher than bees originating from strong colonies in the summer, fall, and early winter (p < 0.01). All weak colonies could not survive the cold weather and died before February. 

### 2.2. Elevated DWV Titers in Cold-Stressed Honey Bees

**Figure 2:**
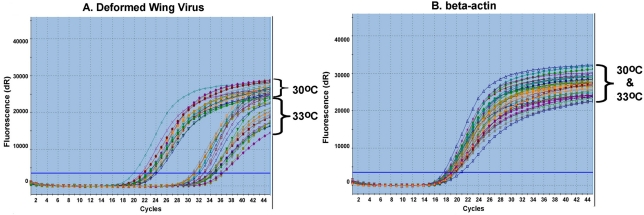
The representative qRT-PCR amplification plots of Deformed Wing Virus (DWV) (**A**) and β-actin (**B**).

A nest temperature of about 33–35 °C was reported to be optimal for honeybee brood development [33,34]. Brood incubated at 29^o^C failed to emerge. The reduction in temperature from 33 °C to 30 °C was accompanied by the addition of four extra days to the 12 day period normally required for pupae to emerge as adults, and a 15% decrease in total number of adult bees that emerged. Further, the quantification of DWV showed that the titer of the virus in newly emerged adult bees from 30 °C incubation was 296 ± 12.57 fold higher than bees from 33 °C incubation. The expression of housekeeping gene β-actin reflects the similar amount of starting material across samples (Figures 2A,B).

### 2.3. Elevated DWV Titers in Varroa-Challenged Bees

Real time RT-PCR showed that DWV levels were higher in bees that originated from both strong and weak colonies after *Varroa *mite challenge. While DWV was also detected in some of the bees collected at day 0 without transferring into rearing cups and exposure to *Varroa* mites, this negative control group had the lowest level of DWV infection among all examined groups and therefore was chosen as a calibrator. The virus level in other groups was expressed as an n-fold difference relative to the calibrator, the negative control group. 

**Figure 3:**
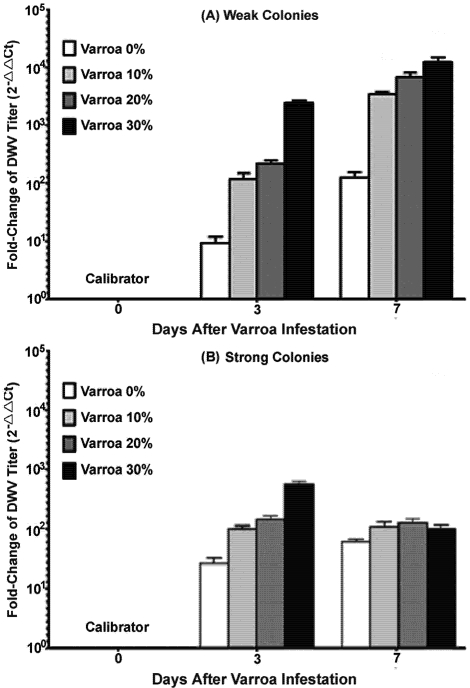
Relative quantitation of the DWV load in bees challenged by *Varroa *mites. (**A**) Bees originated from weak colonies. (**B**) Bee originated from strong colonies. Bees from strong and weak colonies were inoculated with variable levels of *Varroa* mites (0%, 10%, 20% and 30%) to induce the DWV infection and then compared the titers of the virus at different times after *Varroa *mite challenge. The bees collected at day 0 without transferring into rearing cups and exposing to *Varroa* mites were used as a negative control. While DWV was also detected in some bees in the negative control group, these had low levels of DWV infection. The virus level in other groups was expressed as an n‑fold difference relative to the negative control group.

For bees taken from weak colonies (Figure 3A), the DWV titer steadily increased over the whole experiment following the initial *Varroa *mite introduction and reached a maximum at day 7 post‑treatment. The highest DWV titer was observed in the group of bees with 30% *Varroa* mite parasitism at day 7 post treatment. There was a significant positive correlation between the dose of DWV and number of *Varroa* mites introduced at each time point following *Varroa *mite exposure (R^2^ = 0.82 at day 3 and R^2^ = 0.99 at day 7; two-way ANOVA F = 5.059, p = 0.0171).

For bees taken from strong colonies (Figure 3B), DWV concentration showed an increase, and a positive correlation between the dose of DWV and the level of mite parasitism, similar to the profile observed in bees from weak colonies (R^2^ = 0.88; two-way ANOVA F = 11.74, p = 0.0013). However, following the initial increase of DWV at the early time-point post *Varroa* mite infestation (Day 3), the DWV levels declined at the later time-point of *Varroa *infestation (day 7). Additionally, the titers of DWV did not differ significantly across groups challenged by different levels of *Varroa *mites at day 7 post *Varroa *exposure (R^2^ = 0.66), as seen in bees from weak colonies.

### 2.4. Vitellogenin Expression

**Figure 4:**
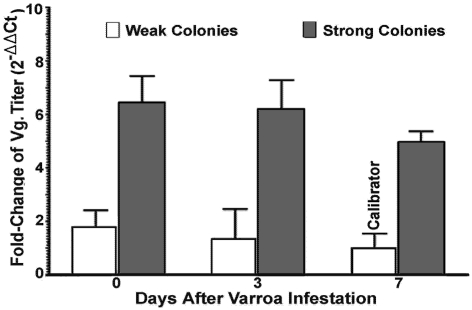
Relative quantitation of Vitellogenin (Vg) transcripts in bees from strong and weak colonies after *Varroa *mite infestation.

The present study confirmed our previous study’s findings showing that the vitellogenin transcript level in bees originating from strong colonies was significantly higher than bees from weak colonies prior to *Varroa* challenge [35], and further demonstrated that the gene expression of vitellogenin in bees was independent from the levels and time period of *Varroa* mite infestation and DWV infection. No difference in vitellogenin gene expression across groups with different levels of *Varroa* mites for bees from both strong and weak colonies at different time points post *Varroa* exposure was observed (day 3 R^2^ = 0.51; day 7 R^2^ = 0.48). For the sake of clear illustration, the relative expression of vitellogenin obtained at different time points post *Varroa* mite exposure at each category was pooled. While the expression of vitellogenin in each category of bees was not correlated with levels of *Varroa* mite challenges, a significant difference in the titer of the vitellogenin was observed between bees from strong and weak colonies (Figure 4) (two-way ANOVA F = 12.15; p = 0.0011). 

## 3. Experimental Section

### 3.1. Honey Bee Colonies and Varroa Mites

**Figure 5:**
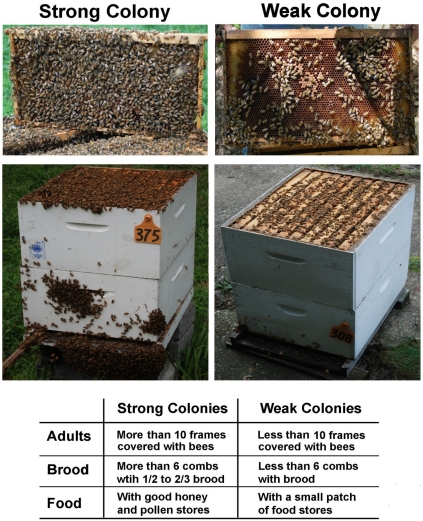
Demonstration of comparatively strong and weak colonies in the study.

Honey bee colonies were maintained in two different USDA Bee Research Laboratory apiaries in Beltsville, MD. The *Varroa* mites were monitored by the natural drop detection method and treated as needed to ensure that mite levels remain below damaging levels throughout the study. The strength of the honey bee colonies was determined based on the number of adult bees and brood as well as the amount of food stored in the combs. The bee colonies that had more than ten frames covered with adult workers and more than six frames filled with brood and food stores were defined as strong colonies, while bee colonies that had a small number of foraging bees flying in and out, less than ten frames of adult bees, less than six combs with brood and small patches of food stores were defined as weak colonies (Figure 5). The colonies that were heavily infested by *Varroa* mites were left untreated and used as *Varroa* resource colonies. They were maintained in a separate apiary. 

### 3.2. Field Investigation of DWV Seasonal Prevalence in Colonies

To assess the seasonality and prevalence of viral infections of honey bees in nature, five strong colonies and five weak colonies were chosen for virus field screening studies. Samples of 25 bees from each colony were collected every month for a period of one year starting in March and finishing in February of the following year. All field samples were individually subject to RNA extraction and RT‑PCR analysis for DWV and other common bee viruses (data not shown). The specificity of PCR products was confirmed by sequencing analysis. The monthly variation of DWV infection, in terms of the percentage of viral infected individuals present in each colony was recorded each month. 

### 3.3. Laboratory Temperature Stress Experiment

The experiments to evaluate the virus titer change in response to cold stress were focused on bees originating from strong colonies since our investigation of virus seasonal activities and colony survivorship showed that all weak colonies could not survive the winter in Maryland. To obtain worker bees of uniform age, the queens from strong colonies were individually confined in a brood nest for 24 hours to lay eggs. After brood cells were capped, the brood comb was divided into three nearly equal parts with each piece containing around 100 capped brood cells. The cells were transferred into emergence cages and kept individually in incubators set at 29 °C, 30 °C or 33 °C. The length of time required for pupa development and numbers of emergent adults were recorded. The emerged adults were removed from the cages and subjected to subsequent RNA isolation and qRT-PCR analysis for DWV. The procedure was repeated three times. 

### 3.4. Laboratory Varroa-Challenging Experiment

The laboratory experiments were conducted during a period between spring and summer, in the peak brooding season when bees were actively foraging. Frames with emerging brood were removed individually from four strong and four weak colonies, placed in separate mesh-walled cages and incubated in an insect growth chamber at 33 °C, 70% RH overnight. Emerging bees were collected the following day and separated into two different categories based on the conditions of the colonies they originated from, strong or weak. Each category was further divided into four treatment sub-groups based on levels of *Varroa* mites introduced. Thirty bees for each group were transferred to a ‘top‑feeder’ rearing cup [36] and variable levels of *Varroa* mites were then introduced: 0% (N = 0), 10% (3 mites per 30 bees), 20% (6 mites per 30 bees), and 30% (9 mites per 30 bees). One subgroup of 10 newly emerged bees from strong and weak colonies were collected individually prior to exposure to cage isolation stress and *Varroa* mite challenge to serve as negative controls and defined as day 0 in the study. Half of the bees along with their *Varroa *mites were collected three days after *Varroa* exposure and the other half of the bees were collected at day 7. 

### 3.5. RNA Extraction

Total RNA was isolated by placing bees in a microcentrifuge tube individually and homogenizing 1 mL of TRIzol Reagent (RNA extraction kit, Invitrogen, Carlsbad, CA, USA) according to the manufacturer’s instructions. Following precipitation and centrifugation, the resultant RNA pellets were re-suspended in UltraPure DNase/RNase-free distilled water (Invitrogen, Carlsbad, CA, USA) in the presence of Ribonuclease Inhibitor (Invitrogen, Carlsbad, CA, USA). The concentration of total RNA was determined by measuring the absorption at 260 nm and purity of RNA was estimated by the absorbance ratio of 260 nm / 280 nm using spectrophotometer (Ultrospec 3300 *pro*, Amersham Biosciences). RNA samples were stored at −80 °C freezer until used. 

### 3.6. Primers and Probes

Sequences of primers and probe specific for DWV and a housekeeping gene, *A. mellifera* β-actin, were reported previously [17]. A previously published primer pair for detection of gene transcript levels of vitellogenin [37] was employed for measurement of general robustness of honey bees in this study. All the primers were synthesized by Invitrogen and the TaqMan probes were purchased from Applied Biosystems. 

### 3.7. TaqMan Real-Time Quantitative RT-PCR (qRT-PCR) for DWV

The quantification of DWV titer in individual bees was performed by TaqMan real time qRT-PCR using a Stratagene Mx3005P^TM^ Multiplex Quantitative PCR System. The amplification was carried out using the one-step Access RT-PCR System (Promega, Madison, WI) incorporated with a 0.2 μM TaqMan probe. The amplification profile consisted of one cycle of 48 °C for 45 min followed by 40 cycles at 95 °C for 30 s, 55 °C for 1 min, and 68 °C for 2 min. The amplification of β-actin was performed for all of the samples analyzed to normalize the results. The final PCR product was confirmed by 1.5% agarose gel electrophoresis with ethidium bromide. Negative controls (no template) were included in each run of the reaction and yielded no products.

The DWV titers in bees were determined based on the value of the cycle threshold (Ct), which represents the number of cycles needed to generate a fluorescent signal above a predefined threshold and therefore is inversely proportional to the concentration of the initial target that has been amplified. The Ct values of the PCRs from each subgroup were averaged and relative quantification of DWV titers was performed using the comparative Ct method, also referred to as the ΔΔ*C*_T_ method. Briefly, the average amount of DWV titer of each group was normalized to internal control β-actin following the formula: ΔCt = Ct_DWV_ − Ct_β-actin_. The Δ*C*_t_ value of each group was relative to a calibrator which represented the minimal virus level following the formula ΔΔCt = Δ*C*_t(target)_ − Δ*C*_t(calibrator)_. The fold‑change in the DWV concentration of each treatment group was calculated using the formula 2^−ΔΔCt^. The comparative CT method was validated by showing that the amplification efficiencies of DWV and β-actin were equal. 

**Figure 6:**
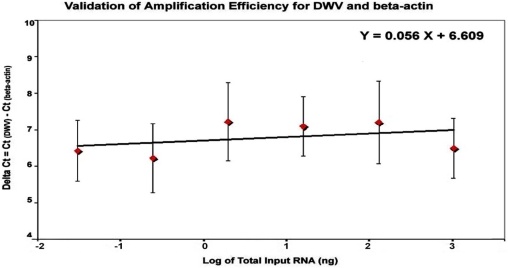
Validation of amplification efficiency for DWV and β-actin by TagMan qRT‑PCR. The amplification of six five-fold dilutions of total RNA ranging from 1 μg to 0.32 ng per reaction was performed in triplicate and the standard curves for DWV and β‑actin were generated by plotting the C_T_ value against the corresponding input RNA. The difference between the C_T_ value of DWV and that of β-actin (ΔCt) was plotted against the log of the corresponding amounts of input RNA.

The comparative C_T_ method was used for quantification of DWV titers. In order to qualify the use of the comparative C_T_ method for DWV quantification, the amplification efficiencies of the TaqMan real time qRT-PCR assay for both DWV and β-actin need to be approximately equal. The amplification of six five-fold dilutions of total RNA ranging from 1μg to 0.32 ng per reaction was performed in triplicate and the standard curves for DWV and β-actin were generated by plotting the C_T_ value against the corresponding input RNA. A linear relationship was observed between the amount of input RNA and the C_T_ values for both DWV and β-actin. The coefficient of correlation (R*^2^*), which indicates the linearity of the C_T_ values plotted in the standard curves, was 0.9822 and 0.9572 for DWV and β-actin, respectively. To control for variation in RNA samples, the concentration of DWV in all tested samples was normalized to the housekeeping gene, β-actin (ΔC_T_). Relative efficiencies of DWV and β-actin amplification were plotted as ΔCt = (C_T(DWV)_ − C_T(β-actin)_) *versus* the log of the corresponding amounts of input RNA. As demonstrated in Figure 6, the efficiency plot between log input RNA and ΔCt had a slope less than 0.1 (slope = 0.056), indicating that efficiencies of DWV and β‑actin amplification were approximately equal (Figure 6). Therefore, the comparative *C*_T_ for relative quantification of DWV in bees challenged by different levels of *Varroa* mites was valid. 

### 3.8. SYBR-Green Real-Time qRT-PCR for Vitellogenin

The quantification of vitellogenin in bees from strong and weak colonies was performed using SYBR Green real-time qRT-PCR. RT-PCR reactions were carried out in a 50-µL reaction volume containing 25 µL of 2× Brilliant® SYBR® Green QRT-PCR Master Mix (Stratagene, La Jolla, CA, USA), 0.4 µM forward and reverse primers, and 1ug of template RNA. The thermal profile for the one step RT-PCR was as follows: one cycle at 50 °C for 30 minutes, one cycle at 95 °C for 10 min followed by 30 cycles of 95 °C for 30 s, 55 °C for 1 min, and 72 °C for 30 s. SYBR Green dye may cause non-specific binding because it intercalates with any double-stranded DNAs and emits fluorescence signals. Therefore, a melting curve analysis was performed after amplification to determine the specificity of the PCR products. The PCR products were incubated for 1 minute at 95 °C, ramping down to 55 °C at a rate of 0.2 °C/s followed by 81 cycles of incubation where the temperature was increased by 0.5 °C/cycle, beginning at 55 °C and ending at 95 °C. The amplification of β-actin, was also performed for each samples to normalize the result. Negative controls (no reverse transcriptase and no template) were included in each run of the reaction and yielded no products. The interpretation and validation of SYBR-Green Real-time qRT-PCR data for vitellogenin quantification were conducted using the comparative Ct method as described above. 

**Figure 7:**
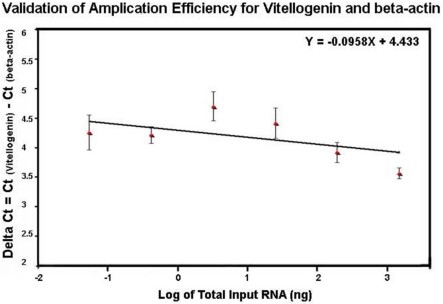
Validation of amplification efficiency for vitellogenin and β-actin by SYBR Green qRT-PCR. The amplification of six five-fold dilutions of total RNA ranging from 1 μg to 0.32 ng per reaction was performed in triplicate and the standard curves for vitellogenin and β-actin were generated by plotting the C_T_ value against the corresponding input RNA. The difference between the C_T_ value of vitellogenin and that of β-actin (ΔCt) was *versus* the log of the corresponding amounts of input RNA.

The profile of the melting curve showed the presence of a single homogeneous melt peak for all sample reactions, confirming specific amplification of vitellogenin primers used in the study (data not shown). The same method used for comparing efficiencies of DWV and β-actin amplification was also used for evaluation of efficiencies of vitellogenin and β-actin amplification. The relative efficiencies of vitellogenin and β-actin amplification by plotting ΔCt = Ct_vitellogenin_ − Ct _β-actin_ *versus* the log of the corresponding amounts of input RNA had a slope less than 0.1 (slope = 0.0958) (Figure 7), indicating that efficiencies of Vitellogenin and β-actin amplification were approximately equal and that the comparative *C*_T_ method for relative quantification of vitellogenin in this study was applicable.

### 3.9. Statistical Analysis

All determinations were made at least in triplicate and the results, in terms of Ct values are expressed as mean ± standard deviation (S.D.). A two-sample Student's t-test was used to analyze for significant differences of DWV infection rate between bees originating from strong and weak colonies each month and the DWV titers between bees incubated into two different temperatures (30 °C *versus* 33 °C). Differences were considered statistically significant if p value < 0.05.

In order to obtain a distribution of the experimental data much closer to the normality [33], the titer of DWV as well as Vg transcripts were log transformed for statistical analysis. Normality of data was checked using the Shapiro-Wilk test and D’Agostino-Pearson test, while homoscedasticity was checked with Bartlett’s test using the statistical package *Prism 5 for Mac OS X*, ver. 5.0c; GraphPad software; San Diego, California, USA. A two-way ANOVA was used to compare genome equivalents of the DWV and Vg considering three factorial levels used in pairs: the *Varroa* mite inoculation (0, 10, 20, 30%), the day post-treatment (3 *vs.* 7) and the type of colony (weak *vs.* strong). With the purpose to measure the strength of the linear relationship, the coefficient of correlation, R^2^ [38], was calculated between the *Varroa* mite inoculation and both viral copies and Vg copies.

## 4. Conclusions

DWV is one of the most common and prevalent viruses found in honey bees. Previous studies showed that DWV usually persists as an inapparent infection in bee colonies via both vertical and food-borne transmission pathways but infection of DWV can multiply to lethal levels to cause severe colony mortality in association with *Varroa *mites [39]. While significant progress has been made in elucidating the genomic structure and transmission process of DWV infection, very little is known about the mechanisms that regulate the persistent and acute infection in bees. In particular, host factors in the pathogenesis of DWV infection have not been extensively explored. This paper describes field and laboratory studies to examine the role of biotic and abiotic factors and host conditions on the replication and progression of DWV in honey bees. Our results clearly indicate that stress factors and host health conditions could have a profound influence on the dynamics of the virus replication and the outcome of the host-pathogen interactions. 

The investigation of the virus’s seasonal activities showed that a long-term persistent infection of DWV has been established in honey bees. Inapparent DWV-infected bees were readily identified in bee colonies examined. However, there was a variation in bees’ resistance to DWV infections in different seasons. The infection of DWV is more common in fall and winter. This variation in frequency likely depends on the host’s strength and weather conditions that modulate normal development and functions of the hosts. 

The observation of the significantly higher titer of DWV in bees from temperature-challenged bees suggests that cold-stress could not only cause a delay in normal brood development but also weaken the host immune response and increase susceptibility of hosts to viral infection. The weaker bee colonies that do not have sufficient bees to maintain a strong cluster for heat generation and retention create an increased vulnerability to virus infection and therefore easily succumbed to cold weather and collapsed rapidly in the early winter. Bee colonies with strong populations of healthy bees and adequate food stores have better regulation for brood temperature. However, significant temperature fluctuations during the winter could also alter colonies’ abilities to regulate immune response and control infection, leading to an elevated virus titer and an increased severity of the virus infection and eventually impacting the bees’ survival. The results from our study partly explains the high levels of winter losses associated with cold stress and support the previous finding that DWV could act independently of *Varroa* mites to lead to colony losses [40]. 

It has long been recognized that *Varroa *mites are effective vectors and activators of several honey bee viruses including DWV. Not surprisingly, increasing the number of *Varroa* mites had the effect of enhancing the concentration of DWV in bees. The presence of DWV in newly emerged adult bees prior to the transmission assay indicated the persistence of DWV in honey bee colonies likely via virus vertical transmission pathway [18]. The accelerating effect of *Varroa* mite feeding on the rate of group infection and titer of DWV in individual adults observed in this study confirmed a previous finding that *Varroa *mites increase the infectivity of DWV not only by its ability to serve as a vector for horizontal transmission from infected individuals to uninfected individuals, but also as an activator of the virus within the hosts. However, host conditions were also found to play a role in the outcome of the host-parasite-pathogen interactions. During the course of the experiment, bees from weak colonies were more susceptible to virus infection by having a significantly higher level of virus load compared to bees from strong colonies. Additionally, while a continuously elevated virus titer was found in bees from weak colonies, there was a decline in virus titer at the later phase of the experiment in bees from strong colonies, after an initial increase in response to *Varroa *mite challenge. This result suggests the ability of host immunity to clear viruses and other immune complexes. This survival advantage displayed in bees originating from strong colonies indicates that host factors played an important role in determining the susceptibility and immune responses of honey bees to virus infection.

There was a significant difference in the level of vitellogenin between bees from strong and weak colonies, although expression of vitellogenin was not reflected by any difference in the level of virus replication and degree of *Varroa* mite infestation. Vitellogenin is a yolk precursor protein and synthesized depending on pollen consumption in honey bees [41]. Vitellogenin is normally secreted into the hemolymph of honey bee queens before being transported into developing oocytes, and accounts for over 50% of total hemolymph proteins in queens [42]. However, vitellogenin is also synthesized by the sterile honey bee workers [43,44]. Aside from having a reproductive function, it has been suggested that vitellogenin serves important functions related to the immunity and longevity of honey bees [37,44,45,46,47]. It was also shown that honey bees use vitellogenin to produce royal jelly [48]. Although vitellogenin is an important host component affecting robustness, it is not a component of the immune pathways. Therefore, the expression of vitellogenin in this study was found not to be influenced immediately in response to the stresses caused by DWV and *Varroa* mites. It is more likely that vitellogenin levels reflect the physiological status of the host and that physiological robustness in turn leads to a spectrum of immune responses in bees from strong colonies against pathogenic and parasitic invasion. The physiological immunity and social immunity of social insects have been gaining increased attention in recent years and has been suggested to be reasons for the relatively few innate immune genes found in honey bees compared with the fruit fly *Drosophila melanogaster* and the mosquito *Anopheles gambiae* [49]. In view of the data presented in this paper, it is clear that the physical status of honey bees has a profound effect on host immunocompetence to resist virus transmission, replication, and disease progression, and that beekeeping practices that promote all aspects of bee health and keep queens well-mated and vigorous at all times can be a key area to target in protecting against pathogenic and parasitic threats.

In sum, our study showed that honey bee host conditions and stress factors that are involved in the vulnerability to infection seem likely to play a key role in virus replication dynamics. The findings from this study have important implications in enhancing our understanding of host-pathogen interactions and provide an explanation for varying degrees of virulence of virus infections in the field and suggest that beekeeping management should focus on minimizing stress as much as possible to keep colonies healthy and strong. Our next step will be to use genome-scale approaches to identify and characterize genes of the host that are actively involved in affecting the progression and outcome of diseases to achieve a better understanding of the mechanisms of host responses to virus infections, which may ultimately lead to new tools for inhibiting virus replication through targeting the host and thereby mitigating viral disease impacts on honey bee populations

## References

[1] Gallaiab N., Sallesc J.-M., Setteled J., Bernard E., Vaissièrea B.E. (2009). Economic valuation of the vulnerability of world agriculture confronted with pollinator decline. Ecol. Econ..

[2] vanEngelsdorp D., Underwood R., Caron D., Hayes J.J. (2007). An estimate of managed colony losses in the winter of 2006–2007: A report commissioned by the Apiary Inspectors of America. Am. Bee J..

[3] Neumann P., Carreck N.L. (2010). Honey bee colony losses. J. Apic. Res..

[4] Cox-Foster D.L., Conlan S., Holmes E., Palacios G., Evans J.D., Moran N.A., Quan P.L., Briese T., Hornig M., Geiser D.M. (2007). A metagenomic survey of microbes in honey bee colony collapse disorder. Science.

[5] van Engelsdorp D., Evans J.D., Saegerman C., Mullin C., Haubruge E., Nguyen B.K., Frazier M., Frazier J., Cox-Foster D., Chen Y.P. (2009). Colony collapse disorder: A descriptive study. PLoS One.

[6] van Engelsdorp D., Meixner M.D. (2010). A historical review of managed honey bee populations in Europe and the United States and the factors that may affect them. J. Invertebr. Pathol..

[7] Allen M., Ball B. (1996). The incidence and world distribution of honey bee viruses. Bee World.

[8] Ellis J.D., Munn P.A. (2005). The worldwide health status of honey bees. Bee World.

[9] Bailey L., Carpenter J.M., Woods R.D. (1981). Properties of a filamentous virus of the honey bee (*Apis mellifera*).. Virology.

[10] Bromenshenk J.J., Henderson C.B., Wick C.H., Stanford M.F., Zulich A.W., Jabbour R.E., Deshpande S.V., McCubbin P.E., Seccomb R.A., Welch P.M. (2010). Iridovirus and microsporidian linked to honey bee colony decline. PLoS One.

[11] Olivier V., Blanchard P., Chaouch S., Lallemand P., Schurr F., Celle O., Dubois E., Tordo N., Thieèy R., Houlgatte R. (2008). Molecular characterization and phylogenetic analysis of chronic bee paralysis virus, a honey bee virus. Virus Res..

[12] van Engelsdorp D., Speybroeck N., Evans J., Nguyen B.K., Mullin C., Frazier M., Frazier J., Cox-Foster D., Chen Y.P., Tarpy D.R. (2010). Identification of risk factors associated with bee colony collapse disorder by classification and regression tree analysis. J. Econ. Entomol..

[13] Bailey L., Ball B.V. (1991). Honey Bee Pathology.

[14] Antúnez K.D., Alessandro B., Corbella E., Ramalllo G., Zunino P. (2006). Honeybee viruses in Uruguay. J. Invertebr. Pathol..

[15] Chen Y.P., Nakashima N., Christian P., Bonning B.C., Valles S.M., Lightner D.V., King A.M. (2011). Iflaviridae. Virus Taxonomy, Ninth Report of the ICTV.

[16] Lanzi G., de Miranda J.R., Boniotti M.B., Cameron C.E., Lavazza A., Capucci L., Camazine S.M., Rossi C. (2006). Molecular and biological characterization of deformed wing virus of honey bees (*Apis mellifera* L.).. J. Virol..

[17] Chen Y.P., Higgins J.A., Feldlaufer M.F. (2004). Quantitative analysis of deformed wing virus infection in the honey bee, *Apis mellifera* L. by real-time RT-PCR. Appli. Environ. Microbiol..

[18] Chen Y.P., Pettis J.S., Collins A., Feldlaufer M.F. (2006). Prevalence and transmission of honey bee viruses. Appli. Environ. Microbiol..

[19] de Miranda J.R., Fries I. (2008). Venereal and vertical transmission of deformed wing virus in honeybees (*Apis mellifera* L.).. J. Invertebr. Pathol..

[20] Möckel N., Gisder S., Genersch E. (2011). Horizontal transmission of deformed wing virus: Pathological consequences in adult bees (*Apis mellifera*) depend on the transmission route. J. Gen. Virol..

[21] Yue C., Gisder S., Genersch E. (2007). Vertical-transmission routes for deformed wing virus of honeybees (*Apis mellifera*).. J Gen Virol..

[22] Ball B.V., Bailey L., Morse R.A., Flottum K. (1997). Viruses. Honey Bee Pest, Predators, and Diseases.

[23] Kovac H., Crailsheim K. (1988). Life span of *Apis mellifera Carnica* Pollm. Infested by *Varroa jacobsoni* in relation to season and extent of infestation. J. Api. Res..

[24] Ball B.V., Allen M.F. (1988). The prevalence of pathogens in honey bee (*Apis mellifera*) colonies infested with the parasitic mite *Varroa jacobsoni*. Ann. Appl. Biol..

[25] Bowen-Walker P.L., Martin S.J., Gunn A. (1999). The transmission of deformed wing virus between honey bees (*Apis mellifera* L.) by the ectoparasitic mite *Varroa jacobsoni* Oud. J. Invertebr. Pathol..

[26] Martin S., Hogarth A., van Breda J., Perrett J. (1998). A scientific note on *Varroa jacobsoni* Oudemans and the collapse of *Apis mellifera* L. colonies in the United Kingdom. Apidologie.

[27] Nordström S. (2003). Distribution of deformed wing virus within honey (*Apis mellifera*) brood cells infected with the ectoparasitic mite *Varroa destructor*. Exp. Appl. Acarol..

[28] Nordström S., Fries I., Aarhus A., Hansen H., Korpela S. (1999). Virus infections in Nordic honey bee colonies with no, low or severe *Varroa jacobsoni* infections. Apidologie.

[29] Shen M.Q., Yang X.L., Cox-Foster D., Cui L.W. (2005). The role of *Varroa* mites in infections of Kashmir bee virus (KBV) and deformed wing virus (DWV) in honey bees. Virology.

[30] Tentcheva D., Gauthier L., Jouve S., Canabady-Rochelle L., Dainat B., Cousserants F., Colin M.E., Ball B.V., Bergoin M. (2004). Polymerase chain reaction detection of deformed wing virus (DWV) in *Apis mellifera* and *Varroa destructor*. Apidologie.

[31] Tentcheva D., Gauthier L., Zappulla N., Dainat B., Cousserans F., Colin M.E., Bergoin M. (2004). Prevalence and seasonal variations of six bee viruses in *Apis mellifera* L. and *Varroa destructor* mite populations in France. Appl. Environ. Microbiol..

[32] Yang X., Cox-Foster D.L. (2005). Impact of an ectoparasite on the immunity and pathology of an invertebrate: Evidence for host immunosuppression and viral amplification. Proc. Natl. Acad. Sci. U. S. A..

[33] Heinrich B. (1980). Mechanisms of body-temperature regulation in honeybees, *Apis mellifera*. J. Exp. Biol..

[34] Winston M. (1987). The Biology of the Honey Bee.

[35] Di Prisco G., Pennacchio F., Emilio C., Boncristiani H.F., Evans J.D., Chen Y.P. (2011). *Varroa destructor* is an effective vector of Israeli acute paralysis virus in the honeybee, *Apis mellifera*. J. Gen. Virol..

[36] Evans J.D., Chen Y.P., Prisco G.D., Pettis J., Williams V. (2009). Bee cups: Single-use cages for honey bee experiments. J. Api. Res..

[37] Corona M., Velarde R.A., Remolina S., Moran-Lauter A., Wang Y., Hughes K.A., Robinson G.E. (2007). Vitellogenin, juvenile hormone, insulin signaling, and queen honey bee longevity. Proc. Natl. Acad. Sci. U. S. A..

[38] Zar J.H. (2009). Biostatistical Analysis.

[39] Chen Y.P., Siede R. (2007). Honey bee viruses. Adv. Virus Res..

[40] Highfield A.C., Nagar A.E., Mackinder L.C.M., Noël L.M-L.J., Hall M.J., Martin S.J., Schroeder D.C. (2009). Deformed wing virus implicated in overwintering honeybee colony losses. Appli. Environ. Microbiol..

[41] Raikhel A.S., Dhadialla T.S. (1992). Accumulation of yolk proteins in insect oocytes. Ann. Rev. Ent..

[42] Hartfelder K., Engels W. (1998). Social insect polymorphism: hormonal regulation of plasticity in development and reproduction in the honey bee. Cur. Top. Devel. Biol..

[43] Byrne B.M., Gruber M.A.G. (1989). The evolution of egg yolk proteins. Prog. Biophy. Mol. Biol..

[44] Seehuus C., Norberg K., Gimsa U., Krekling T., Amdam G.V. (2006). Reproductive protein protects functionally sterile honey bee workers from oxidative stress. Proc. Natl. Acad. Sci. U. S. A..

[45] Amdam G.V., Aase A.L., Seehuus S.C., Fondrk M.K., Norberg K., Hartfelder K. (2005). Social reversal of immunosenescence in honey bee workers. Exp. Gerontol..

[46] Amdam G.V., Csondes A., Fondrk M.K., Page R.E. (2006). Complex social behaviour derived from maternal reproductive traits. Nature.

[47] Amdam G.V., Simões Z.L.P., Hagen A., Norberg K., Schrøder K., Mikkelsen Ø., Kirkwood T.B.L., Omholt S.W. (2004). Hormonal control of the yolk precursor vitellogenin regulates immune function and longevity in honeybees. Exp. Gerontol..

[48] Amdam G.V., Norberg K., Hagen A., Omholt S.W. (2003). Social exploitation of vitellogenin. Proc. Natl. Acad. Sci. U. S. A..

[49] Honey Bee Genome Sequencing Consortium (2006). Insights into social insects from the genome of the honey bee *Apis mellifera*. Nature.

